# An Effective Treatment for Tinnitus and Hyperacusis Based on Cognitive Behavioral Therapy in an Inpatient Setting: A 10-Year Retrospective Outcome Analysis

**DOI:** 10.3389/fpsyt.2020.00025

**Published:** 2020-02-07

**Authors:** Daniele R. Nolan, Rahul Gupta, Christian G. Huber, Andres R. Schneeberger

**Affiliations:** ^1^Department of Adult Psychiatry, Psychiatric Services of Grisons, Chur, Switzerland; ^2^Department of Adult Psychiatry, University Psychiatric Clinics, Basel, Switzerland; ^3^Department of Psychiatry, Psychitherapy and Psychosomatics, Psychiatric University Clinic, Zurich, Switzerland; ^4^Department of Psyhiatry and Behavioral Sciences, Albert Einstein College of Medicine, New York, NY, United States

**Keywords:** tinnitus, hyperacusis, treatment, cognitive behavioral therapy, efficacy

## Abstract

**Aim:**

Tinnitus and hyperacusis are phenomena with a considerable prevalence in the general population, leading to high levels of suffering. It is a symptom that can present itself comorbidly with a variety of psychiatric and medical illnesses. We established a treatment of tinnitus and hyperacusis, which is based on a multimodal approach including a specific cognitive behavioral therapy (CBT) method in an inpatient setting. This approach includes education on tinnitus and hyperacusis, applying coping strategies and techniques of relaxation, directed attention, and music therapy. We aim to evaluate the efficacy of this treatment approach.

**Materials and Methods:**

We included retrospective data of 268 patients who underwent tinnitus treatment throughout the 10-year existence of the treatment program. We assessed routine clinical data pretreatment and posttreatment with parameters concerning tinnitus–distress, hyperacusis, and psychological well-being. To determine these variables, we used validated instruments including the Tinnitus Questionnaire (TQ), Questionnaire on Hypersensitivity to Sound (QHS), Brief Symptom Inventory (BSI), and the Beck Depression Inventory (BDI-II).

**Results:**

Patients showed highly significant reduction in all of the examined clinical outcomes. Reduction of TQ, the primary outcome measure, was 15.39 (SD 21.88) from a mean baseline value of 35.72 (p < 0.001). The QHS showed a reduction of 6.72 (SD 8.23) from a mean baseline value of 18.98 (p < 0.001). Moreover, psychological strain was also reduced with high significance, as illustrated in reduction of BSI and BDI-II; reduction in BSI from 49.63 by 24.41 (SD 26.88; p < 0.001) and BDI-II from 16.89 by 7.47 (SD 8.76; p < 0.001).

**Discussion:**

The multimodal treatment program for tinnitus and hyperacusis including a specific CBT method proves to be a highly effective means of significantly reducing not only tinnitus and hyperacusis but also accompanying distress. Furthermore, it also enables considerable reduction of concomitant psychiatric symptoms such as depression.

**Conclusions:**

Our results underline the importance of intensive and multimodal approaches to the treatment of tinnitus and hyperacusis.

## Introduction

Tinnitus is the perception of sound in the ears or head unrelated to an external source (1). In Europe, prevalence is estimated at 21.2% (2),while prevalence in Switzerland is described at a similar level with 20% (3). Individuals above the age of 55 years show higher rates than younger individuals (1). Of persons with tinnitus, 7.9% experience frequent tinnitus, which involves perceptions at least once daily (4). Tinnitus perceived as severe has a prevalence of 1.3% (1).

Evidence concerning rates of tinnitus related to age and gender varies greatly in the literature; age and gender seem to have an influence, although there is no agreement as to how (1, 2, 4). Udupi et al. (5) found tinnitus severity to have a significant correlation with depression and anxiety symptoms, while they found no significant correlation between tinnitus severity and age or gender. Younger men (< 50 years) seem to experience the least emotional distress (depression and anxiety), followed by older men (> 50 years) (6).

Although the severity of tinnitus is associated with anxiety and depression, causality cannot definitely be concluded (7). In addition to depression and anxiety, there is a higher incidence of somatoform disorders in individuals with tinnitus (8). Among further comorbidities associated to tinnitus is hyperacusis, defined as an increased sensitivity to ordinary environmental sounds that would not usually be bothersome to most individuals (9). The threshold for this uncomfortable loudness level is set at <100 dB for all frequencies (10). Estimations suggest that 40% to 55% of patients with tinnitus comorbidly suffer from hyperacusis (11). Conflicting data regarding hyperacusis in relation to gender exist; while one study found hyperacusis to be more common among females (12), other data found it to be more frequent among males (13, 14). Also, people with self-reported hyperacusis are younger and have higher education than people whose hyperacusis was diagnosed by a physician (12). Furthermore, people with hyperacusis and tinnitus tend to be younger than those with only tinnitus. There is strong evidence that hyperacusis has a positive correlation with tinnitus loudness and depressive symptoms (14), as well as with depression, anxiety, and functional somatic syndromes (12).

Treatment options for tinnitus solely achieve amelioration; no medical cures for tinnitus exist so far (15, 16). Tinnitus masking and tinnitus retraining therapy are two successfully applied methods (17). Tinnitus masking uses wideband noise applied through ear-level devices to the point that provides greatest relief from tinnitus, accompanied by counseling on the appropriate use of ear-level devices and general advice about tinnitus (15). In contrast, in tinnitus retraining therapy, ear-level sound is adjusted to the point where it starts to mix together with the tinnitus, accompanied by a structured counseling method (17).Although both methods show remarkable improvement in ameliorating tinnitus, in comparison to tinnitus masking, the effects of tinnitus retraining therapy improve progressively over time (17). Also, the results of tinnitus retraining therapy sustain over time (18) and show to improve quality of life of those affected (19). In recent years, there have been numerous publications implementing a therapeutic model based on cognitive behavioral therapy (CBT), which is applied in different countries worldwide (20). It has been shown that CBT for tinnitus is effective irrespective of tinnitus severity (15), which reduces tinnitus symptoms significantly (21). Depression and overall psychiatric symptoms are improved (22). Achieved amelioration persists up to 15 years following treatment (23), whereby it tends to continuously improve in the first year after treatment (24). While CBT protocols usually take several weeks, it has been shown that tinnitus retraining therapy carried out as a short-term therapy (e.g., 1 week) is effective at the end of the program, as well as maintaining the achieved results over time (24). Whereas most treatments focus on group therapy, individual CBT shows to also be effective (25).

While the literature presents a lot of studies about the impact of tinnitus on psychiatric symptoms (8) and how treatment might impact these comorbidities, less has been published about the effects of hyperacusis. Studies have shown that hyperacusis is related to tinnitus loudness, discomfort, and annoyance as well as depressive symptoms (14). However, no significant correlation between the presence of hyperacusis and tinnitus annoyance could be found (13). Also, researchers showed in their analysis that hyperacusis has a high comorbidity with psychiatric diagnoses and somatic syndromes (12). Up to 56% of hyperacusis patients were found to have at least one current psychiatric disorder, of which 47% had an anxiety disorder (26).

Tinnitus patients in Switzerland are treated in an inpatient setting at the psychiatric services of Grisons since January 2006. A multimodal treatment program based upon CBT as a modified tinnitus retraining therapy is employed. The specifics of the treatment program have already been published shortly after initiation of the program (27), as well as preliminary evaluations with 56 study subjects, which showed promising data (28).

With this study, we aim to evaluate the efficacy of the aforementioned treatment program for tinnitus over a time span of 10 years with a retrospective analysis of routine clinical data. Our goal is to show that the treatment program is effective in treating tinnitus and hyperacusis as well as comorbid depressive symptoms and the consequential general symptom intensity. Apart from evaluation of treatment efficacy we additionally aim to investigate the phenomenon of hyperacusis in regard to its relationship to gender, as hereof conflicting data exist.

We hypothesize that the implemented therapy program is effective in the treatment of tinnitus. Next to effectively treating tinnitus, we hypothesize that the treatment program is also effective on treatment of concomitant hyperacusis as well as depressive symptoms and accompanying general symptom intensity. Effective treatment of tinnitus and its comorbidities could lead to a significant improvement in many aspects of the life of affected people, which from our point of view is an essential purpose of medicine.

## Methods

### Study Design and Participants

This single-center study was conducted in the Psychiatric Services Grisons, Waldhaus clinic in Chur, Canton Grisons, Switzerland, between March 2006 and December 2015. The clinic network includes 230 psychiatric inpatient beds and has the state mandate for mental health care of the Canton Grisons. The study's catchment area is characterized by lack of major cities (populations over 50,000); the major business sectors include tourism and farming. The Canton Grisons is entirely alpine (41% of the people live at 3,000 feet or higher), and the population density is low (68.8 persons per square mile). Also, the rate of psychiatrists is lower compared to the rest of Switzerland, with 0.15 practitioners per 1,000 inhabitants (Swiss average: 0.26 psychiatrists per 1,000 residents) (29). Patients were referred by an ear-nose-throat specialist (ENT), a general practitioner, a family physician, a psychiatrist, or by themselves, which constitutes a clinical convenience sample.Inclusion criteria included present tinnitus and/or hyperacusis. Exclusion criteria were present conditions subsumed under the categories “Organic, including symptomatic, mental disorders,” “Mental and behavioral disorders due to psychoactive substance abuse and dependency” (except Tobacco), or “Schizophrenia, schizotypal and delusional disorders“ according to the International Classification of Diseases version 10 (ICD-10) (30), as well as inability to read or write German (**Table 1**). The study was approved by the local Swiss ethics committee (KEK-ZH BASEC Nr. 2016-00620) and conducted according to the principles expressed in the Declaration of Helsinki (31). The study was registered in the U.S. NIH clinical study protocol registration system (appointed the Identifier Number NCT02632058 on ClinialTrials.gov).

**Table 1:** Inclusion and exclusion criteria.

Inclusion criteria	Exclusion criteria
Present tinnitus and/or hyperacusis	Organic disorders, including symptomatic, mental disorders
Age 18 or older	Mental and behavioral disorders due to psychoactive substance abuse and dependency (except tobacco)
	Schizophrenia, schizotypal and delusional disorders
	Inability to read or write German

### Treatment Concept

Before treatment, each patient underwent audiological assessment by an ENT to evaluate individual hearing threshold (pure tone audiometry varying from 0.125 to 16 kHz), uncomfortable loudness levels, and tinnitus masking frequency. In cases of hearing loss, which is strongly associated with tinnitus (1), hearing aids were implemented.

Treatment protocol was applied equally to all subjects and lasted for 6–8 weeks. In case of comorbid psychiatric illnesses, standard treatment protocols were administered by the treating physicians who were unrelated to the study. The following steps consisted of individual and group therapy as listed in **Table 2**.

**Table 2:** Implemented therapeutic modalities.

-Psychoeducation on tinnitus and hyperacusis
-Cognitive restructuring	
-Musical and hearing therapy
-Application of directed attention and mindfulness techniques
-Progressive muscle relaxation
-Ear acupuncture
-Physical therapy of the shoulder-neck-jaw region
-Relaxation techniques by use of creative therapy
-Training of emotional competencies
-Training of social competencies
-Weekly individual therapy with psychiatrist

Initially, patients underwent psychoeducation concerning tinnitus and hyperacusis. With individual or group psychoeducation, subjects are supplied with established scientific knowledge on tinnitus and hyperacusis in order to better understand an illness and its treatment. Cognitive restructuring is a psychotherapeutic method and essential tool in CBT to identify and rearrange maladaptive thoughts about a specific subject (e.g., tinnitus and hyperacusis) and can be applied individually or in a group. Directed attention and mindfulness techniques are techniques applied in CBT with the aim of better coping with psychiatric illnesses and stressful situations. Progressive muscle relaxation is a relaxation technique used in CBT where muscle groups are deliberately and alternatively tensed up and then relaxed, causing a state of deep relaxation. Physical therapy (i.e., massage and physiotherapy) of the shoulder-neck-jaw region and ear acupuncture, where thin needles are inserted in the ear, also aim at relaxing tightened up muscles and inducing a state of relaxation. Creative therapy in CBT practices the use of art, dance, movement, and music to promote development and healing. Through training of emotional and social competencies in interaction with other individuals within a therapeutic group, those competencies are improved, as they can be reduced in psychiatric patients (32, 33). Weekly individual therapy with a psychiatrist addresses the individual problems, situation, goals, and cognition.

The main components of the therapy protocol were elements of cognitive behavioral psychotherapy (as described above); the focus was laid on individual problems, goals, and cognition of each participant. In order to be successful this procedure requires the willingness to change and collaboration of every individual. With this procedure, therapy participants were put in a position to further change their habits and cognition in their private and professional surroundings to be able to autonomously deal with difficult situations and thus perpetuate a mechanism of capacity building (27).

One other specific treatment modality involved in the treatment program was musical and hearing therapy. The goal hereby is regaining delight in hearing through restructuring of acoustic perception. This happens primarily by changing one's perception on the basis of positive hearing experiences which in turn influence one's hearing and concomitantly one's overall condition. This subsequently enables the transformation of once unpleasant noises into pleasant acoustic inputs (34).

### Measures

All measures were based on self-report questionnaires. Apart from assessment of audiometry, no other objective measures were implemented.

The *Tinnitus Questionnaire* (TQ) is a self-assessment questionnaire consisting of 52 items with three answer possibilities ranging from “true” to “somewhat true” and “not true.” The questions cover six areas in which tinnitus might be a burden: a) emotional impairment, b) cognitive impairment, c) intrusiveness of tinnitus, d) acoustic impairment, e) sleep impairment, f) somatic impairment. Additionally, a global score for tinnitus–distress can be ascertained. Point scores in the TQ ranges from 0 to 84 and are divided into four categories, each signifying a severity of tinnitus (grade 1 = 0–20; grade 2 = 21–46; grade 3 = 47–59; grade 4 = 60–84). A score above 46 points equals a decompensated, that is, debilitating tinnitus. The TQ is a validated instrument with a test–retest reliability of r = 0.94 for the global TQ score and between r = 0.86 and r = 0.92 for each subscale. Internal consistency (Cronbach's alpha) is α = 0.94 for the global TQ score and for the subscale ranges between α = 0.74 and α = 0.92 (35).

The *Questionnaire on Hypersensitivity to Sound* (QHS) is a self-assessment questionnaire consisting of 15 items that capture the subjective impairment through hyperacusis, attitude, as well as behavior and reactions on external sources of sound. Items are answered on a four-point Likert scale (“not true,” “sometimes true,” “often true,” and always true”). Internal consistency ranges from α = 0.77 through α = 0.82 (36).

The *Brief Symptom Inventory* (BSI) collects subjective impairment through somatic and psychological symptoms over the last 7 days. The items can be grouped into nine scales consisting of somatization, compulsion, insecurity, depression, anxiety, aggression, phobic anxiety, paranoid thoughts, and psychoticism. Internal consistency of the nine scales ranges from r = 0.39 und r = 0.75, depending on the sample. Retest reliability of the global parameter lies within r = 0.92 (37).

The *Beck Depression Inventory* (BDI-II) (38) is a self-assessment tool assessing the severity of depressive symptoms according to DSM-IV. The questionnaire has a good content validity; its internal consistency is α ≥ 0.84 and retest reliability is r ≥ 0.75 in nonclinical samples. Furthermore, the BDI-II distinguishes well between different degrees of depression while maintaining sensitivity to change (39).

### Statistical Methods

Primary endpoint was change of tinnitus severity on the basis of the alteration of the TQ. Secondary endpoints were pretreatment and posttreatment differences in the scores of QHS, BSI, and BDI-II. The significance level was determined at a p-value of 0.05 and including a Bonferroni adjusted p-value of 0.0125. For statistical calculation purposes, we used the Statistical Package for the Social Sciences (SPSS) software (40).

We used a Welch t-test to test for differences in the continuous variables assuming unequal variances of the tested variables. To assess differences in categorical variables, we used chi-squared tests. To analyze the correlation between tinnitus and hyperacusis on depressive symptoms and psychological strain, we constructed a multiple regression model. We used this multiple linear regression to explain the observed dependent variables depressive symptoms and in a second model psychological stress by several independent variables, including tinnitus and hyperacusis. This relationship is overlaid by possible additive confounders. Based on clinical experience and the existing literature, we used gender and age as possible confounding variables.

## Results

### Clinical and Sociodemographic Variables

Over a time span of 10 years, a total of 268 participants (male n = 173 or 64.3% and female n = 95 or 35.4%) were included in this study. All participants met inclusion criteria, and therefore, none were excluded from the study. Also, none of the participants refused treatment.

Average age was roughly 49 years, ranging from 18 to 75 years. Of those patients, two thirds were between the ages of 40 and 59. Men accounted for almost two thirds of all patients and were on average slightly younger than women. Most male participants were between the ages of 40 and 49 years, followed by the category of 50–59 years old. The largest group of female participants was within 50–59 years old, followed by the category of 40–49 years.

About half of all men were married and had completed an apprenticeship, whereas less than half of the women had completed an apprenticeship. Completion of an apprenticeship was the most common educational status in the population. Academic graduation (college/university) was more than three times higher for men than women.

Roughly 95% of the participants were Swiss citizens, while two men were citizens of Lichtenstein and 12 were citizens of other European countries. Differences in age, citizenship, education, or marital status with reference to gender were nonsignificant (**Table 3**).

**Table 3:** Population characteristics.

Study Sample	Male		Female		Total		p-value
	Years	SD	Years	SD	Years	SD	
**Age (Mean)**	47.61	11.39	50.19	12.98	48.53	12.02	0.93
**Age Group**	**N**	**%**	**N**	**%**	**N**	**%**	0.43
under 20	1	0.6	0	0.0	1	0.4	
20–29	10	5.8	6	6.3	1	6.0	
30–39	28	16.2	14	14.7	42	15.7	
40–49	57	32.9	22	23.2	79	29.5	
50–59	55	31.8	27	28.4	82	30.6	
60–69	17	9.8	21	22.1	38	14.2	
70–79	5	2.9	5	5.3	10	3.7	
**Citizenship**	**N**	**%**	**N**	**%**	**N**	**%**	0.267
Swiss	162	93.6	92	96.8	254	94.8	
Liechtenstein	2	1.2	0	0	2	0.7	
Other	9	5.2	3	3.2	12	4.5	
**Educational Status**	**N**	**%**	**N**	**%**	**N**	**%**	0.578
Apprenticeship	95	54.9	40	42.1	135	50.4	
Vocational school	22	12.7	15	15.8	37	13.8	
No graduation	3	1.7	7	7.4	10	3.7	
Higher school certificate	3	1.7	1	1.1	4	1.5	
Grade school	5	2.9	10	10.5	15	5.6	
College or university	24	13.9	4	4.2	28	10.4	
Unknown	21	12.4	18	18.9	39	14.6	
**Marital Status**	**N**	**%**	**N**	**%**	**N**	**%**	0.042
Never married	49	28.3	28	29.5	77	28.7	
Married/Common law	86	49.7	34	35.8	120	44.8	
Widowed	1	0.6	5	5.3	6	2.2	
Divorced	20	11.6	15	15.8	35	13.1	
Separated	3	1.7	0	0	3	1.1	
Unknown	14	8.1	13	13.6	27	10.1	

Data are n (%) or mean (SD), unless noted otherwise. Categorical values were compared with chi-squared test and metric variables with t-test.

SD, standard deviation.

Fifty (18.7%) individuals exhibited hyperacusis in addition to tinnitus; 24 (8.9%) of those were women, while 26 (9.7%) were men. Mean age was 48.4 years (SD 12.3), and most were married and had completed an apprenticeship. Individuals with hyperacusis were slightly younger compared to those with tinnitus alone (48.4 vs. 48.6).

### Tinnitus, Hyperacusis Severity, and Psychological Strain

**Table 4** presents an overview on tinnitus, hyperacusis severity, and psychological strain at admittance and at discharge.

**Table 4:** Difference of scores in employed questionnaires pre and post study.

	TQ	QHS	BSI	BDI-II
**Pre**	35.72	18.98	49.63	16.89
**Post**	20.32	12.26	25.21	9.41
**Difference**	15.39	6.72	24.41	7.47
**SD**	21.88	8.23	26.88	8.76
**p-value^a^**	<0.001	<0.001	<0.001	<0.001
**Cohen's d**	0.70	0.82	0.79	0.74

TQ, Tinnitus Questionnaire.

QHS, Questionnaire on Hypersensitivity to Sound.

BSI, Brief Symptom Inventory.

BDI-II, Beck Depression Inventory II.

SD, standard deviation of the coefficient.

a Data were compared with t-test.

Reduction of symptoms was significant in all employed questionnaires; tinnitus severity especially was significantly reduced according to the TQ. Additionally, significant reduction of hyperacusis (QHS) and psychological strain was ascertained as illustrated in reduction of BSI and BDI-II. Symptom reduction of the TQ subscales is presented in **Table 5**.

**Table 5:** Differences of Tinnitus Questionnaire (TQ) subscales.

	E^a^	C^b^	I^c^	A^d^	SI^e^	SO^f^
**Pre**	10.19	6.46	9.06	4.57	3.49	1.94
**Post**	5.24	3.52	5.65	2.84	1.95	1.13
**Difference**	4.94	2.94	3.41	1.74	1.54	0.81
**SD^g^**	6.68	4.42	5.39	3.52	2.60	1.75
**p-value^h^**	<0.001	<0.001	<0.001	<0.001	<0.001	<0.001

a E, emotional impairment.

b C, cognitive impairment.

c I, intrusiveness of tinnitus.

d A, acoustic impairment.

e SI, sleep impairment.

f SO, somatic impairment.

g SD, standard deviation of the coefficient.

h Data were compared with t-test.

### Predictors of BSI and BDI-II Score Changes

A multiple regression was performed to predict the difference between the BDI-II score at baseline and at discharge from age, gender, tinnitus at baseline, and tinnitus difference. The multiple regression model statistically significantly predicted BDI-II F(4, 152) = 12.278, p < 0.0005, adj. R^2^ = 0.224. Only tinnitus difference added statistically significantly to the prediction, p < 0.05. Regression coefficients and standard errors can be found in **Table 6**. In a second step, we added hyperacusis at baseline and hyperacusis to the regression model. It statistically significantly predicted BDI-II F(6, 131) = 17.260, p < 0.0005, adj. R^2^ = 0.416. Tinnitus difference and hyperacusis difference added significantly to the prediction, p < 0.05.

**Table 6:** Summary of multiple regression analysis: BDI-II difference.

	B^a^	SE^b^	Beta^c^	T^d^	p-value
Model 1
Intercept	-7.967	3.156		-2.525	0.013
Age	0.087	0.051	0.121	1.713	0.089
Gender	-0.212	1.292	-0.012	-0.164	0.87
Tinnitus at baseline	0.017	0.042	0.035	0.408	0.684
Tinnitus difference	0.22	0.038	0.491	5.762	<0.001

**Model 2**					
Intercept	-2.997	3.119		-0.961	0.338
Age	0.059	0.048	0.08	1.213	0.227
Gender	-1.142	1.213	-0.062	-0.942	0.348
Tinnitus at baseline	-0.058	0.052	-0.109	-1.117	0.266
Hyperacusis at baseline	0.134	0.083	0.15	1.608	0.11
Tinnitus difference	0.117	0.052	0.234	2.234	0.027
Hyperacusis difference	0.549	0.105	0.501	5.218	<0.001

a B, unstandardized regression coefficient.

b SE, standard error of the coefficient.

c Beta, standardized regression coefficient.

d T, t-test.

**Table 7** shows a multiple regression to predict the difference between the BSI score at baseline and at discharge from age, gender, tinnitus at baseline, and tinnitus difference. The multiple regression model statistically significantly predicted BSI F(4, 107) = 6.553, p < 0.0005, adj. R^2^ = 0.167. Only tinnitus difference added statistically significantly to the prediction, p < 0.05. Adding hyperacusis at baseline and hyperacusis difference, the multiple regression model statistically significantly predicted BSI F(6, 178) = 5.642, p < 0.0005, adj. R^2^ = 0.249. Tinnitus difference and hyperacusis difference added statistically significantly to the prediction, p < 0.05.

**Table 7:** Summary of multiple regression analysis: BSI difference.

	B^a^	SE^b^	Beta^c^	T^d^	p-value^e^
Model 1					
Intercept	-21.279	12.319		-1.727	0.087
Age	0.127	0.213	0.052	0.596	0.552
Gender	6.967	4.915	0.124	1.417	0.159
Tinnitus at baseline	-0.085	0.151	-0.063	-0.562	0.576
Tinnitus difference	0.457	0.13	0.392	3.519	0.001

**Model 2**					
Intercept	-12.151	15.283		-0.795	0.429
Age	0.243	0.239	0.096	1.014	0.314
Gender	0.494	5.57	0.009	0.089	0.93
Tinnitus at baseline	-0.268	0.213	-0.165	-1.259	0.212
Hyperacusis at baseline	0.276	0.358	0.101	0.772	0.443
Tinnitus difference	0.455	0.204	0.295	2.229	0.029
Hyperacusis difference	0.97	0.441	0.295	2.199	0.031

a B, unstandardized regression coefficient.

b SE, standard error of the coefficient.

c Beta, standardized regression coefficient.

d T, t-test.

e Data were compared with t-test.

## Discussion

The age distribution showed that most participants were between 40 and 60 years old, which corresponds to a common prevalence documented in the literature (4).The population in our study showed no gender difference in all sociodemographic variables except in marital status. Men were significantly more often married, with marriage being the most often occurring marital status in the population. This corresponds to the current literature, where most people with tinnitus are married, although irrespective of gender (23). This is possibly due to the fact that men have a shorter life expectancy than women, so that women tend to outlive their husbands and therefore shift the weight from the “married” category onto the “widowed” category.In one study, we found that individuals with tinnitus had 13–15 years of education (1); in a second study, most patients had an education below the level of high school (4), as well as in a third study, where the level of education of tinnitus patients was mostly found to be “low” (15). In comparison, in our study population, most individuals completed an apprenticeship, which can vary from 12 to 15 years of education in total. Most had an education above the level of high school, and only about one in 10 had an education considered to be “low.”It is difficult to compare educational status among studies due to the international variation of school systems. Educational level of our sample is relatively high, which is most likely due to Switzerland having a well-developed educational system.

The implemented multimodal treatment program for tinnitus and hyperacusis, including a specific CBT method with musical therapy, different relaxation techniques, and directed attention, shows to be a highly effective means of treatment; impairments due to tinnitus and hyperacusis were both significantly reduced. All included outcome measures, such as the BDI-II to assess depressive symptoms, showed a significant difference between the values at baseline compared to the assessment before discharge from the clinic. The analysis of the subjective symptom intensity as measured by the BSI also demonstrated a clear reduction throughout the course of treatment, which confirms prior findings (15, 18, 21, 24, 25). Our results regarding reduction of depressive symptoms support prior studies stating that the reduction of depressive symptoms is an often found outcome in the treatment of tinnitus (22, 23). In summary, our results support earlier studies (15, 18, 19, 22, 24, 25) that a treatment specifically geared at tinnitus in addition to psychiatric treatment as usual benefits people suffering from tinnitus.

In our population, we found a higher prevalence of hyperacusis in the female population, which contrasts earlier studies (13, 14, 41, 42). Individuals with hyperacusis were in fact slightly younger compared to those with tinnitus, although the age difference was much smaller compared to similar findings (14). Furthermore, other studies showed an increased prevalence of hyperacusis with age (43), a finding we could not replicate in our population.Our multiple regression model showed an increase of the coefficient of determination (r^2^) when adding the reduction in hyperacusis (**Tables 4** and **5**). According to our results, reduction of tinnitus alone and reduction of tinnitus and hyperacusis significantly account for the variability in depression. The analysis showed that additionally targeting hyperacusis might increase the chances of a successful treatment outcome, especially in regard to somatic and psychological symptoms. This seems very plausible when considering higher tinnitus-related distress, general distress, and decreased mental and somatic health in patients with hyperacusis and tinnitus in comparison to patients with tinnitus alone (14).

A limitation of this study is that only retrospective data were used, which limits determination of effects on outcome and does not allow causal explanations for the observed associations. In addition, as a clinical convenience sample, selection bias might have been relevant. On the other hand, all admitted patients were included in the study and completed treatment, with no dropouts. Due to the naturalistic design of the study, subjects were either admitted by an ENT, a general practitioner, a family physician, a psychiatrist, or by themselves if they suffered from tinnitus. There were no explicit criteria for being admitted (e.g., tinnitus severity). Nevertheless, naturalistic studies are considered to be useful study designs that approach real clinical settings and do not lose ecological validity due to randomization.

Administration of psychopharmacological treatment might limit validity of treatment effectiveness, although there is still insufficient evidence to underline antidepressant drug therapy to improve tinnitus (44). In essence, the effects of medication in this study remain unclear, although evidence suggests *Ginkgo biloba* extract having an effect on the treatment of tinnitus (45). No control group for comparison was included, which lies within the naturalistic study design where we offer treatment to any referred patient. Although the study shows our treatment program to be effective, there is no differentiation as to which one of the single elements involved in our multimodal treatment program is predominant in treating tinnitus and/or hyperacusis effectively.

In conclusion, we were able to show that the multimodal treatment program for tinnitus might be a highly effective means of not only treating tinnitus and hyperacusis but also targeting comorbid psychiatric symptoms. We were able to show that focusing on the treatment of hyperacusis can have an additional benefit in reducing psychiatric symptoms. Considering the scarcity of studies targeting hyperacusis, we point out that future research should integrate the effects of hyperacusis and the possible benefits of specifically targeting hyperacusis in the treatment of tinnitus.

## Data Availability Statement

The datasets generated for this study are available on request to the corresponding author.

## Ethics Statement

The studies involving human participants were reviewed and approved by Kanton Zuerich Kantonale Ethikkomission. The patients/participants provided their written informed consent to participate in this study.

## Author Contributions

All authors designed the study. AS and DN submitted the request for approval to the ethics committee. DN wrote the initial draft of the paper. All authors analyzed and interpreted the data and contributed to reading and approving the final version of the manuscript. All authors had full access to all the data in the study and take responsibility for the integrity of the data and the accuracy of the data analysis.

## Conflict of Interest

The authors declare that the research was conducted in the absence of any commercial or financial relationships that could be construed as a potential conflict of interest.
